# The Changing Prevalence of Pressure Injury among Ontarians with SCI/D at Rehabilitation Admission: Opportunities for Improvement

**DOI:** 10.3390/healthcare12111084

**Published:** 2024-05-25

**Authors:** Beverley Catharine Craven, Emma A. Bateman, Heather Flett, Farnoosh Farahani, Dalton L. Wolfe, Sussan Askari, Maryam Omidvar, Mohammad Alavinia

**Affiliations:** 1 Lyndhurst Centre, The KITE Research Institute, Toronto Rehabilitation Institute, University Health Network, Toronto, ON M4G 3V9, Canada; cathy.craven@uhn.ca (B.C.C.); heather.flett@uhn.ca (H.F.); farnoosh.farahani@uhn.ca (F.F.); maryam.omidvar@uhn.ca (M.O.); 2 Temerty Faculty of Medicine, University of Toronto, Toronto, ON M5S 1A8, Canada; 3 Parkwood Institute Research, St Joseph’s Health Care London, London, ON N6A 4V2, Canada; ali.bateman@sjhc.london.on.ca (E.A.B.); dwolfe@uwo.ca (D.L.W.); 4 Department of Physical Medicine & Rehabilitation, Schulich School of Medicine & Dentistry, Western University, London, ON N6A 5C1, Canada; 5 Faculty of Health Sciences, School of Health Studies, Western University, London, ON N6A 5C1, Canada; 6 Faculty of Physical Medicine and Rehabilitation, Providence Care Hospital, Queen’s University, Kingston, ON K7L 4X3, Canada; askaris@providencecare.ca

**Keywords:** spinal cord injury, pressure injury, daily skin check, health system

## Abstract

Background: Despite preventability, 20–50% of patients with acute spinal cord injury/disease (SCI/D) develop hospital-acquired pressure injuries (PIs). The Spinal Cord Injury Implementation and Evaluation Quality Care Consortium (SCI IEQCC) aimed to mitigate PI risk through patient-reported daily skin checks alongside usual care. Methods: This quality improvement initiative utilized an interrupted time series design, encompassing adults ≥ 18 years admitted for inpatient rehabilitation across five Ontario sites from 2020 to 2023. Patient demographics, etiology, and impairment data were obtained from a national registry, while participating sites gathered data on PI onset, location, and severity. Run charts depicted temporal trends, and statistical analyses, including chi-square and logistic regression, compared patients with and without PIs. Results: Data from 1767 discharged SCI/D patients revealed that 26% had ≥1 PI, with 59% being prevalent and 41% incident. Most severe PIs (stages III and IV and unstageable) were acquired prior to admission. Process indicator fidelity was reasonable at 68%. Patients with PIs experienced longer hospital stays, lower Functional Independence Measure (FIM) changes, and FIM efficiency during rehabilitation. Conclusions: PI prevalence is increasing, particularly sacral injuries at admission, while incident cases have decreased since 2021 due to regular skin checks. This trend calls for proactive health system interventions to reduce costs and improve patient outcomes.

## 1. Introduction

Neither the term pressure injury (PI) nor its predecessors, pressure wound, pressure ulcer, or bed sore, convey the devastating health and health system impacts of this condition. Health complications attributable to PIs include serious infections such as cellulitis and or osteomyelitis, impaired participation in rehabilitation due to bed rest or pressure-relieving activities, increased pain, reduced health-related quality of life, prolonged hospital length of stay (LOS), and increased healthcare costs [1]. Thus, PI prevention is of the utmost importance.

Persons with spinal cord injury/disease (SCI/D) are particularly vulnerable to developing PIs due to the motor, sensory, bowel, bladder, and autonomic impairments associated with spinal cord dysfunction. Immobility, the loss of sensation, moisture, and shear forces increase the risk of PI development. Despite being preventable, 20–50% of persons with acute SCI/D will develop one or more PIs during their hospitalization [2,3]. Further, 85–95% of persons with SCI/D will develop at least one PI over their lifetime [4,5].

PI preventive strategies are well established [6] and include appropriate bed and seating surfaces, such as low-air-loss mattresses, coupled with frequent repositioning to mitigate immobility; frequent self- or clinician-assisted visual skin examinations to address the lack of sensation; comprehensive bowel and bladder management effectively reduces moisture; and appropriate spasticity management, positioning, and transfer techniques effectively mitigate shear stresses. Despite evidence that the best practices prevent PIs, patients hospitalized with SCI/D experience PIs due to isolated or combined health systems’ failures to provide appropriate surfaces, frequent repositioning, a visual inspection of skin, and timely care to mitigate moisture and reduce shear stressors in acute settings and inpatient rehabilitation settings.

The Spinal Cord Injury Implementation and Evaluation Quality Care Consortium (SCI IEQCC) or Consortium aims to provide optimal and equitable care for Ontarians to improve the quality of rehabilitation care for all persons living with SCI/D, regardless of where they live, including preventing PIs and optimizing care within the domain of Tissue Integrity [7]. The Consortium, a province-wide initiative amongst five tertiary SCI/D rehabilitation hospitals, started in January 2019 and implemented indicators for tracking optimal care within this domain of care in January 2020.

Among the five participating SCI/D specialty rehabilitation hospitals, health system leaders reported a perceived surge in PI prevalence amongst persons admitted for SCI/D rehabilitation during the COVID-19 pandemic and a health services mandate issued by the provincial Ministry of Health (MOH) in response to unprecedented health system pressures from COVID-19 [8,9]. As tracking PI prevalence at rehabilitation admission and PI incidence during rehabilitation is vital for optimizing patient flow, improving the effectiveness of care, and achieving skin integrity, the Consortium conducted a province-wide analysis of Consortium data from 2020 to 2023 within the Tissue Integrity domain with the following aims: (1) to describe PI prevalence, anatomic location and severity at admission to SCI/D rehabilitation; (2) to describe PI incidence, anatomic location, and severity acquired during inpatient SCI/D rehabilitation; (3) to identify patient factors associated with PI development; (4) to describe how health system flow pressures may be influencing PI prevalence; and (5) to identify opportunities to reduce PI prevalence and PI incidence in hospitalized persons with SCI/D.

## 2. Materials and Methods

Descriptions of the procedures for selecting Tissue Integrity as a priority domain for implementation within the Consortium [10], the Spinal Cord Injury Rehabilitation Care High-Performance Indicators (SCI-HIGH) project methods [11] describing the Consortium’s process for developing indicators and a related evaluation framework, the development of the SCI-HIGH Tissue Integrity indicators [7], and the process of the concurrent implementation of the best practices and collection of structure, process, and outcome indicators within the Consortium [12] are provided in the referenced manuscripts.

### 2.1. Institutional Research Ethics Board Approval

This quality improvement (QI) initiative was undertaken by health system leaders, clinicians, and patients affiliated with five participating tertiary SCI/D rehabilitation sites in Ontario. Research Ethics Board waivers and/or QI approvals were obtained for the project, and appropriate data-sharing agreements and confidentiality agreements were established between participating sites per their institutional requirements: the University Health Network in Toronto (UHN QI # 20-0111), Parkwood Institute in London (REB exemption #116615), and appropriate institutional exemptions from Hamilton Health Sciences in Hamilton, Providence Care in Kingston, and the Ottawa Hospital Rehabilitation Centre in Ottawa.

### 2.2. Project Scope

Data were collected for all adult patients aged ≥ 18 years discharged from inpatient SCI/D rehabilitation at the five Ontario participating sites between January 2020 and July 2023. Patient-level data were de-identified using a unique Consortium ID linked to the individual’s National Rehabilitation Reporting System (NRRS) data. Indicator data were collected at participating sites and then securely transferred to UHN, where the data were pooled, stored, and analyzed. The QI project leader and central team members are responsible for analysis and reporting of data on behalf of the Consortium.

### 2.3. Data Acquisition: Demographic and Rehabilitation Data

In Ontario, all adults (aged ≥ 18 years) with traumatic or non-traumatic SCI/D admitted for inpatient SCI/D rehabilitation are included in the NRRS, a national database of adult inpatient rehabilitation clients [13]. Data collected within the NRRS include patient age, sex, weight, height, the date of rehabilitation admission and discharge, and SCI/D-related neurological impairments: paraplegia/tetraplegia, completeness of injury (complete/incomplete), American Spinal Injury Association (ASIA) Impairment Scale (AIS) category (A, B, C, or D), and admission and discharge Functional Independence Measure (FIM) scores. All patient’s NRRS data were linked to their Consortium ID for this QI initiative. Additional demographic and rehabilitation data were calculated for each patient from the available data. For example, body mass index (BMI), was calculated for each patient by dividing the weight in kilograms by the square of the height in meters using the patient’s weight and height at discharge. When a patient’s height or weight at discharge was unavailable, weight and height at admission were substituted. LOS was calculated by subtracting the rehabilitation discharge from the admission date and then subtracting the days the patient was outside the rehabilitation center for any reason (i.e., medical assessment or emergency room visit). FIM change was calculated for each patient by subtracting the discharge FIM from the admission FIM. FIM efficiency was calculated by subtracting the admission FIM score from the discharge FIM score and dividing this value by LOS. The Consortium counts patients by the number of discharges in the reference period.

### 2.4. Definition and Classification of Pressure Injury—Outcome Indicator

PI was operationally defined, as per the National Pressure Injury Advisory Panel (NPIAP), as “localized damage to the skin and underlying soft tissue” [14] and diagnosed and classified by clinicians at participating sites in accordance with institutional practices and established PI staging. The severity of pressure injuries (PI) was defined using the NPIAP grading stages: Stage I, Stage II, Stage III, Stage VI, Unstageable (U), and Deep Tissue Pressure Injury (DTPI). [15]. PI occurrence was defined as either a prevalent or incident case. Patients with one or more PIs at rehabilitation admission were defined as those with prevalent PIs, and patients who developed PIs during rehabilitation were defined as those with incident PIs. Patients could have both prevalent and incident PIs. For this manuscript, only the first PI identified was used to establish PI prevalence and PI incidence. PI onset prior to or during rehabilitation admission, the number of PIs, their anatomic location(s), the severity of PI, and status (open/closed) at discharge were reported.

### 2.5. Process Indicator Data Collection

The process indicator was the proportion of individuals with SCI/D who completed daily head-to-toe skin checks or skin checks. The number of skin checks per day was collected for all patients. A daily skin check card adapted from Spinal Cord Injury Ontario [16] prompted staff and patients by asking, “Did you or someone else check your skin from head to toe today?” Data were recorded as part of routine clinical practice and then collected via chart abstraction at participating sites. The completion of daily skin checks is an established best practice [6]. Skin check on admission to rehabilitation is an established standard practice, which identifies prevalent PIs (those present on admission). Skin checks during the LOS are intended to identify early incident PI and prevent PI progression. Regulated healthcare professionals, predominantly nurses, record skin check completion throughout the patient’s LOS. Patients are encouraged to participate in the skin check process by conducting self-assessments using a hand-held mirror or cellphone with a selfie stick. Responsibility for the conduct of the daily head-to toe-skin checks are transferred from the regulated healthcare provider to the patient or their family caregiver during the rehabilitation LOS. Routine practice of this skill in a supportive environment is intended to support patients leaving the rehabilitation centers with daily skin checks imbedded in their routine as a lifelong practice. The described data elements were implemented as part of local best practices, leading to some variability in data completion.

### 2.6. Statistical Analysis

The raw data and graphical representations were comprehensively examined to ensure data quality. The appropriate parametric and non-parametric statistical tests were used to describe the following: (1) the proportion of patients with and without PIs using chi-square tests; (2) the patient age, impairment, LOS, FIM change, PI prevalence, and anatomic location, severity, and outcome of prevalent PIs (open or healed) at discharge; (3) the frequency of daily skin checks; and (4) the PI incidence and the anatomic locations, severity, and outcome of incident PI (open or healed) at discharge. Run charts were used to examine interrupted time series data for the outcome measures of PI prevalence at admission and PI incidence during rehabilitation over time, as previously described [17]. Run charts were created in Power Business Intelligence Software (Microsoft Corporation, Redmond, WA, USA, Version: 2.127.1080.0).

Non-parametric statistics were used to test the differences in the severity of injury (AIS A-B), etiology of injury (traumatic versus non-traumatic), level of injury (paraplegia versus tetraplegia), and LOS among patients with and without PIs, as well as PI prevalent and incident cases.

Univariate and multiple logistic regression models were run to establish the relationship between demographic (age, sex, BMI) and injury characteristics (etiology of injury, level of injury, AIS category) and PI prior to or during rehabilitation. The odds ratio (OR) and 95% confidence intervals (CIs) were used to measure the association(s). For selecting relevant variables, all statistically significant variables in the univariate model were evaluated in multiple logistic regression analyses. All statistical analyses were conducted using R version 4.3.0 (R Foundation for Statistical Computing, Vienna, Austria), with an alpha error of 0.05 considering two-tailed statistical tests.

## 3. Results

### 3.1. Cohort Description

From 2020 to 2023 inclusively, 1782 patients with SCI/D were admitted across the Consortium participating SCI/D rehabilitation sites; of these, 15 patients did not have data regarding the prevalence or incidence of PI and were excluded from the analysis. The majority of patients (97%) were admitted for initial rehabilitation. The median and interquartile range (IQR) rehab onset days (time from injury onset to rehabilitation admission) for the initial rehabilitation were 24 (38) days. Data on injury etiology and AIS grade were not available for 44 patient and 132 patients with PIs, respectively.

**Figure 1:**
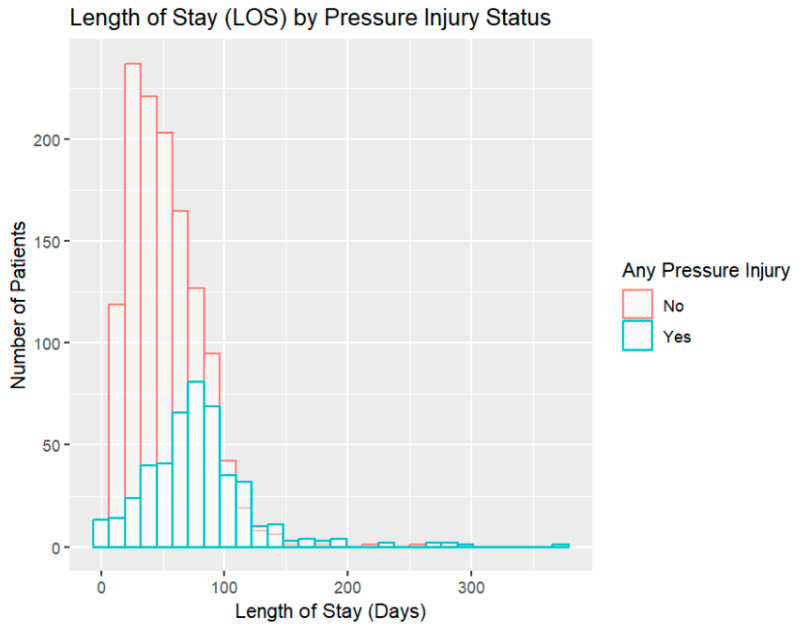
Rehabilitation length of stay (LOS) among patients with and without pressure injury (PI).

Table 1 outlines the characteristics of the remaining 1767 included patients. The average age of included patients was 58.53 ± 16.59 years for males and 60.34 ± 16.59 for females. The majority of patients were male (n = 1060), 38% were female (n = 649), and 60 patients did not disclose their sex. The majority of patients had incomplete paraplegia (50%), followed in descending order of frequency by incomplete tetraplegia (35%), complete tetraplegia (8%), and complete paraplegia (7%). The majority of patients had non-traumatic etiologies of SCI/D (n = 1093, 67%) and motor incomplete injuries (AIS C or D, n = 990, 61%). Figure 1 displays the distribution of LOS among patients with and without PI.

Table 2 shows the characteristics of patients with a PI compared to those without PIs. Patients with at least one PI were slightly older than those without a PI (t_1758_ = −3.2176, *p*-value = 0.001). More males than females had a PI. The analysis showed a slight difference in mean BMI between the groups with and without a PI, with the group with a PI having a slightly higher BMI (t_1552_ = 2.0004, *p*-value = 0.05). Significant univariate associations existed between injury severity and PI: those with AIS A/B impairment were more likely to have PIs than patients with AIS C/D, as discerned via the chi-square test (*p*-value < 0.00001). Patients with at least one PI had a longer LOS in inpatient rehabilitation by 26 days than those without a PI (*p*-value < 0.0001). Patients with PIs experienced a comparatively lower improvement in the FIM compared to patients without PIs (24.4 vs. 30.1, respectively) (*p*-value < 0.0001). The difference in FIM efficiency was even more striking; due to the increased LOS associated with having a PI, FIM efficiency was 0.37 for persons with PIs compared to 0.67 for those without a PI (*p*-value < 0.0001).

### 3.2. Pressure Injury Prevalence, Incidence, and Outcomes

Of the 1767 patients included in the analysis, 275 (15.6%) had at least one PI prior to rehabilitation admission (prevalent PI), and 189 (10.7%) had at least one documented PI during rehabilitation (incident PI). One hundred and twenty-three patients had more than one prevalent PI. Fifty-seven patients had more than one incident PI.

**Figure 2:**
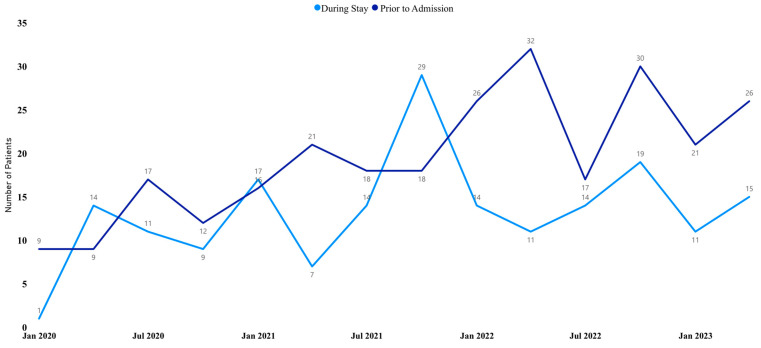
Run chart of annual incidence (during stay) and prevalence (prior to admission) of PIs from January 2020 to July 2023.

The run chart time series analysis from January 2020 to July 2023, indicates prevalent cases are rising. Conversely, the run chart time series analysis over the same time period for incident cases shows these have been declining starting in 2021 (Figure 2).

### 3.3. Skin Checks

**Figure 3:**
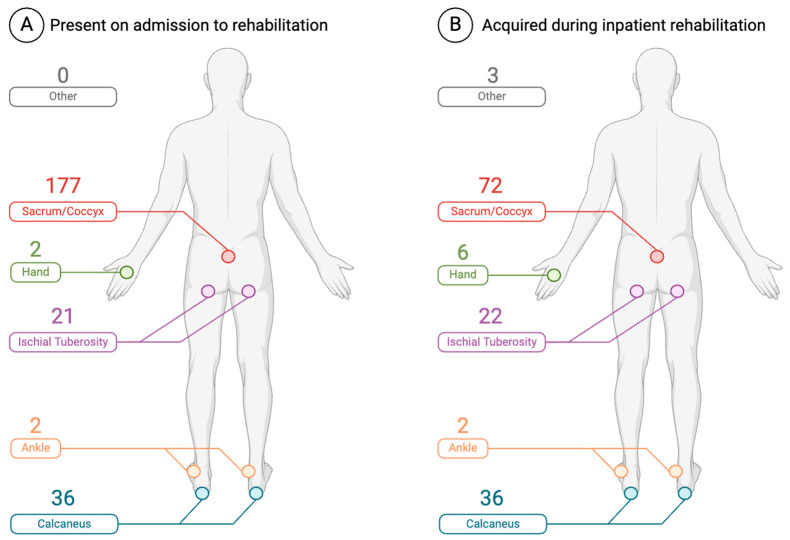
Location and number of (**A**) prevalent and (**B**) incident pressure injuries (created with Biorender.com).

**Figure 4:**
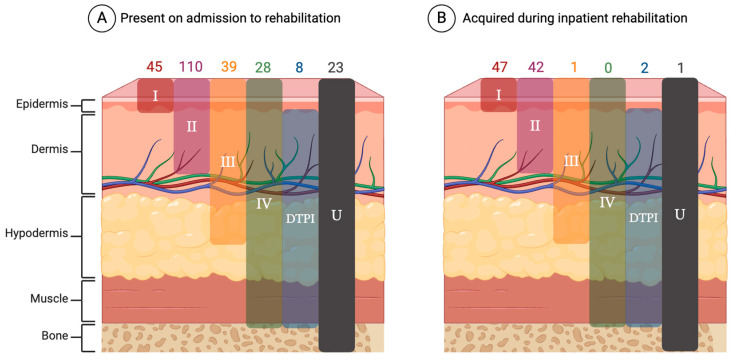
Severity and number of PIs (**A**) PI present on admission to inpatient SCI/D rehabilitation (prevalent cases on the left) and (**B**) acquired PI during inpatient SCI/D rehabilitation (incident cases) (created with Biorender.com). Abbreviations: DTPI—deep tissue pressure injury; U—unstageable.

Process measure data on the frequency of skin checks were available for 1524 patients. Of these, 1032 (67.7%) reported at least one skin check per day. There are significant associations between the stage, location, and status of PIs at rehabilitation admission and the time of occurrence of PIs (before or during rehabilitation). Cases with advanced PI stages (II, III, VI, U, and DTPI) were more common before admission to SCI/D rehabilitation, while less advanced cases (stage I) were more common during the rehabilitation stay (*p*-value < 0.0001) (Table 2). Figure 3 shows the anatomic distribution of PIs by incident and prevalent cases. Figure 4 illustrates the number and severity of PIs by incident and prevalent cases. Information about the PI status (open vs. closed) at discharge were available for 306 cases; of these, 88 (28.8%) were open, and 218 (71.2%) were closed at discharge from the rehabilitation sites (Table 2).

### 3.4. Clinical Factors Associated with Pressure Injury

Table 3 summarizes the outcomes of both univariate and multiple logistic regression. This model was created to establish the relationship between demographic and injury characteristics and the presence of PIs prior to or during rehabilitation. The univariate logistic regression analysis revealed that there are positive associations between the presence of PI and older age, male sex, higher BMI, traumatic etiology, and AIS AB category. After taking into account potential confounding variables using a multiple logistic regression model, age, sex, etiology, and AIS classifications remained statistically significant. Further analysis within the AIS categories revealed that individuals with AIS A/B impairment have 3.86 times greater odds (a 286% higher likelihood) of developing a PI than those with AIS C/D impairment. For age, the odds of developing PIs increased by 3% each additional year of age above age 18 years. While a higher BMI demonstrated a marginal association with PIs in the univariate analysis, this effect became statistically non-significant in the multiple logistic regression.

## 4. Discussion

The Consortium PI data reveal alarming trends in sacral PI prevalence prior to rehabilitation admission and important reductions in PI incidence during inpatient rehabilitation with the implementation of daily skin checks. Stage III and IV and unstageable PIs are healthcare-never events—preventable events that should never happen in healthcare [18]—yet 92 such injuries occurred in persons admitted for inpatient SCI/D rehabilitation. The vast majority of these PIs (n = 90, 98%) occurred prior to rehabilitation admission, a deeply worrying finding associated with current post-pandemic health system pressures. Moreover, the overall prevalence (15.6%) and incidence (10.7%) of PIs for persons with SCI/D are alarming, given the global health system resource dedicated to PI prevention in our province [19]. As PIs are preventable, with appropriate healthcare interventions resulting in a declining incidence, in most acute hospital settings [20], the prevalence and incidence of PIs among patients with SCI/D reflects the inability of the overall health system to adequately meet the care needs of individuals with SCI/D. Prevalent deep sacral and ischial PI are drivers for surgical wound closure in individuals with delayed healing or non-healing PIs [21]. Surgical interventions for wound closure also have inherent risks and associated costs for the individual and health system.

In this QI initiative, patients with SCI/D admitted for inpatient rehabilitation shared similar characteristics to those described in recent epidemiological studies of SCI/D: mostly male patients in older middle age with incomplete injuries [22,23]. However, over 2020–2023, the majority of patients admitted for SCI/D rehabilitation experienced non-traumatic injuries (69.5%), a subpopulation of SCI/D whose epidemiology and related incidence of health complications are less well described in hospital-based studies [23,24,25,26]. The patient factors associated with PIs in this QI initiative mirror those in the existing) literature (predominantly traumatic SCI/D): a greater severity of injury (AIS A/B vs. AIS C/D) was associated with the presence of ≥1 PI [27,28,29,30]; older age was associated with the presence of ≥1 PI [31,32]; and a longer LOS was associated with the presence of ≥1 PI [31]. Male sex and higher BMI were not significantly associated with the presence of a PI, congruent with findings in some studies [30,33] and contrary to findings in other studies [27,34]. The odds of developing a PI are measurably higher for men with motor complete injury and the elderly. The intersection between a longer LOS, reduced FIM efficiency, and PI presence identified in this QI initiative is noteworthy. Patients with a PI had, on average, a 1.5 times longer LOS than patients without a PI. Over that LOS, patients without PI experienced nearly twice the functional improvement, as measured by absolute FIM change (*p*-value < 0.0001). While some of the discrepancy in FIM scores may be explained by patients with PIs having more severe impairments, the complexity of managing PIs is also a likely factor. PIs necessitate meticulous monitoring, expertise in wound care and nutrition, reduced time in a wheelchair, and access to pressure-relieving devices and surfaces, which globally diverts time away from participation in rehabilitation therapy, thereby diminishing functional recovery. Moreover, PI-related infections and pain may further limit a patient’s tolerance for rehabilitation participation. Recognizing this interplay between PIs and rehabilitation outcomes underscores that prevention is paramount.

The observed interplay between PIs, a longer LOS, and reduced FIM efficiency have significant healthcare cost implications. Inpatient SCI/D rehabilitation comprises the bulk of healthcare costs attributable to SCI/D in the first year post-injury, exceeding CAD 120,000 per patient in a historical cohort from 2005 to 2006 [35]. The rehabilitation costs are about three times that of a patient’s acute care stay. Although not measured in this QI initiative, the cost of inpatient SCI/D rehabilitation for persons with PIs exceeds that of patients without PIs, given that their LOS is 1.5 times longer. The incremental increase in the LOS and reduced FIM change associated with PIs suggest patients’ are doubly disadvantaged by a longer LOS in hospital and with less functional improvement. PIs, which are not, resolved prior to discharge from inpatient rehabilitation also incrementally increases the costs of PI care; for community-dwelling persons with SCI/D, the average healthcare cost for PI care is estimated at 4745 CAD monthly for an average of 7 months totaling 33,215 CAD [36].

Future initiatives should explore not only opportunities for improving prevention but also the development of care models prioritizing wound management and functional recovery so that patients with SCI/D who develop PIs are not doubly disadvantaged. The combined incremental costs of inpatient and outpatient care and the adverse impacts on patient outcomes attributable to PIs should be targets of our provincial health authorities, administrators, policy makers, healthcare providers, patients and families alike.

The SCI IEQCC aims to improve the quality of care for persons with SCI/D across Ontario, including reducing PI prevalence and incidence to zero. Rehabilitation hospitals, including participating sites, have tried to achieve this target. However, evidence of effective interventions to reduce PI development is limited [37]. A key intervention deployed in this QI initiative was the implementation of daily skin checks with usual care. The optimal implementation of daily skin checks includes educating patients and or family caregivers about how to complete a daily head-to-toe skin check to confirm the absence of tissue injury over bony prominences using a gooseneck mirror, or cell phone and selfie stick. The inherent assumption is that nurses will initially conduct the daily skin checks and that the responsibility for task completion is incrementally transferred from the nurse, then jointly to the nurse and patient or family caregiver, with gradual transfers of daily head-to-toe skin checks to the patient or their family caregiver with the nurse documenting skin check completion. The Consortium skin check data do not currently distinguish if a regulated healthcare professional, patient, or family caregiver completed the skin check, only that it was conducted.

In our context, the fidelity of implementation [38] is the extent or degree to which the best practices are implemented as intended by the Consortium leadersand SCI-High indicator developers. In principle, simple, specific interventions are more likely to be implemented with high fidelity over complex tasks. Although recording skin check completions, “yes or no”, is relatively simple, conducting the complete skin check and documenting who completed the task is more complex. SCI Ontario and Consortium staff jointly created a video, educating patients on how to conduct a daily head-to-toe skin check to clarify what a skin check entails. The moderate fidelity (68%) of our daily skin checks implies that this task is complex. It recognizes that this is one of many practices at participating sites while acknowledging that skin check implementation has reduced PI incidence and likely reduced PI severity through early detection. Opportunities for further improvement include the following: (1) optimizing the fidelity of this process measure (>90%) in inpatient SCI/D rehabilitation; (2) a high-fidelity translation of this practice upstream in acute care settings where the most severe PI were prevalent; (3) a 1:1 observation of skin checks conduct by skilled staff and peers living with SCI/D may further increase the fidelity of skin checks; and (4) advocacy for health system change.

Disruptions in health system flow arising as a sequelae of the COVID-19 pandemic and related health system pressures may have influenced the high rate of prevalent PIs, including severe PIs, developing prior to admission to inpatient rehabilitation [38,39]. Optimal care for preventing PIs is expertise-, time-, and staff-intensive, all factors that may have been negatively affected by high rates of skilled nursing turnover, higher than typical rates of hospital occupancy, and the increased use of hospital infection control practices that reduce healthcare worker contact with patients [39,40]. The concerning trend of increasing PI prevalence at rehabilitation admission is in part attributable to disruptions in the Ontario SCI model systems, with patients with SCI/D being diverted to community hospitals while awaiting rehab admission, instead of remaining in acute care (i.e., neuro stepdown units) where the staff are educated regarding PI prevention, positioning, the frequency of turning, and the routine use of appropriate air fluidized mattresses and pressure-relieving surfaces.

There is a need for a concerted effort in all healthcare settings to prevent PIs by implementing the best practices [6]. As the adage goes, an ounce of prevention is worth a pound of cure: improved prevention will reduce unnecessary harm to patients with SCI/D and is cost-effective [41].

Limitations of this QI initiative include incomplete data collection, the longtime intervals in the interrupted time series design, and the need for additional root cause analyses to explore the observed trends in PI prevalence and incidence. Some variables, including the conduct of daily skin checks (process indicator) and the status (open/closed) of PIs at discharge, were unavailable in some patients. Moreover, we do not have information about the cause, time of PI onset, duration of PI, or hospital site where the PIs occurred before rehabilitation admission other than the number of sites (trauma center versus community hospital) the patient attended. The large time intervals used in the interrupted time series design limit the understanding of temporal trends [17]. Although the QI methodology offers important health system insights in real-world tertiary SCI/D rehabilitation settings, inferential studies are needed to determine the cause-and-effect relationships between PIs and two or more variables.

## 5. Conclusions

Amongst 1767 patients admitted to five inpatient SCI/D rehabilitation centers, 275 (15.6%) had at least one PI prior to rehabilitation admission, and 189 (10.7%) had a documented new incident PI during inpatient rehabilitation. A vast majority (98%) of the most severe PIs (stage III, IV, and unstageable) were acquired prior to rehabilitation admission, indicating an urgent need to reduce prevalent PI. This QI initiative demonstrates that implementing patient-reported daily skin checks is associated with reduced incidence of PIs in the inpatient rehabilitation setting. Given the magnitude of the consequences of PIs on patients with motor complete injury and health systems, appropriate resources should be allocated to develop innovative prevention strategies, enhance/accelerate PI treatment outcomes, and unpack system-wide opportunities to reduce PI and augment skin integrity.

## Figures and Tables

**Table 1:** Demographic and impairment characteristics of 1767 individuals with SCI/D.

Variable	All Patients	Patients with PI (Prevalent and Incident)	Patients without PI	*p*-Value
	(N = 1767)	(n = 464, 26.3%)	(n = 1303, 73.7%)	
Patient Demographics				
Age—Mean ± SD				0.001
Men	58.53 ± 16.59	60.70 ± 16.00	57.57 ± 16.76	
Women	60.34 ± 16.59	63.24 ± 15.08	16.60 ± 16.89	
Female—n (%)	649 (38%)	132 (29%)	517 (41%)	<0.00001
BMI—mean ± SD	27.58 ± 6.98	27.79 ± 7.18	26.98 ± 6.32	0.045
Etiology of SCI/D *				
Trauma— n (%)	532 (33%)	173 (41%)	359 (30%)	0.0002
Non-trauma— n (%)	1093 (67%)	247 (59%)	849 (70%)	
Severity of SCI/D *				
AIS A, B— n (%)	213(18%)	112 (34%)	101 (12%)	<0.00001
AIS C, D— n (%)	983 (82%)	219 (66%)	764 (88%)	
Rehabilitation Admission Information				
LOS (median (IQR))	55 (44)	74 (40)	47.5 (41)	<0.00001
Mean FIM change (mean ± SD)	28.58 ± 18.59	24.35 ± 19.21	30.09 ± 18.14	<0.00001
FIM efficiency (mean ± SD)	0.59 ± 0.64	0.37 ± 0.68	0.67 ± 61	<0.00001

AIS: American Spinal Injury Association Impairment Scale; BMI: body mass index; FIM: Functional Independence Measure; IQR: interquartile range; LOS: length of stay; PI: pressure injury; SCI/D: spinal cord injury/disease; SD: standard deviation. * Etiology of SCI/D—Trauma: SCI caused by external physical impacts, such as motor vehicle accidents, falls, sports injuries, or acts of violence. Non-trauma: SCI/D resulting from medical conditions or diseases, including tumors, infections, spinal stenosis, or congenital disorders. * The severity of SCI/D is classified using the American Spinal Injury Association (ASIA) Impairment Scale (AIS), which ranges from AIS A (complete injury) to AIS E (normal motor and sensory function). In this study, we combined AIS A and B as one group and AIS C and D as another group and did not consider AIS E.

**Table 2:** Description of pressure injury characteristics among prevalent and incident cases.

	Prevalent PI n = 275	Incident PI n = 189	*p*-Value
PI Anatomic Location—n (%)			
Sacrum	194 (74%)	87 (48%)	<0.00001
Other sites	68 (26%)	93 (52%)	
PI NPIAP Stage—n (%)			
I	45 (18%)	47 (51%)	<0.00001
II	110 (44%)	42 (45%)	
III	39 (15%)	1 (1%)	
IV	28 (11%)	0 (0%)	
Unstageable	23 (9%)	1 (1%)	
DTPI	8 (3%)	2 (2%)	
PI Status at Discharge—n (%)			
Open	62 (34%)	26 (23%)	0.05
Closed	123 (66%)	87 (77%)	

DTPI: deep tissue pressure injury; NPIAP: National Pressure Injury Advisory Panel; PI: pressure injury.

**Table 3:** Univariate and multiple logistic regression for the association between pressure injuries and demographic and injury characteristics.

	Univariate Logistic Regression			Multiple Logistic Regression		
	OR	95% CI	*p*-Value	OR	95% CI	*p*-Value
Age *	1.01	1.00, 1.02	0.001	1.03	1.02, 1.04	<0.0001
Sex—Male	1.73	1.37, 2.18	<0.0001	1.35	0.99–1.85	0.06
BMI *	0.98	0.97, 1.00	0.05	0.98	0.96–1.003	0.11
Traumatic Etiology of Injury	1.65	1.31, 2.08	<0.0001	1.32	0.97–1.80	0.07
AIS–A&B (Motor Complete (Reference = C&D))	3.86	2.81–5.31	<0.0001	5.18	3.56–7.60	<0.0001
Time from Injury to Rehab	1.00	0.99–1.001	0.15			
Intercept	-	-	-	0.06	0.02–0.14	<0.0001

AIS: American Spinal Injury Association Impairment Scale; BMI: body mass index. * Age in years was calculated as the difference between the date of discharge and the date of birth. * BMI was calculated as weight in kilograms divided by height in meters squared (kg/m^2^).

## Data Availability

The data presented in this study are available on request from the project leader and first author, Beverley Catharine Craven.
